# Monkeypox-related knowledge and vaccination willingness among HIV-diagnosed and -suspected males: a cross-sectional survey in Changsha

**DOI:** 10.3389/fpubh.2024.1455623

**Published:** 2025-01-07

**Authors:** Yingying Zhou, Jiemin Wang, Zhi Xie

**Affiliations:** ^1^Changsha Centre for Disease Control and Prevention, Changsha, Hunan, China; ^2^Regenerative Medicine Institute, School of Medicine, University of Galway, Galway, Ireland

**Keywords:** monkeypox, HIV, men who have sex with men (MSM), sexually transmitted infections (STIs), vaccination, health education

## Abstract

**Objective:**

This study aimed to update baseline data on monkeypox (mpox)-related knowledge and vaccination willingness among human immunodeficiency virus (HIV) diagnosed and suspected males.

**Methods:**

The cross-sectional survey was conducted in Changsha, a provincial capital in China, during 5 JULY to 5 SEPTEMBER 2023. Among the three study groups, the participants in the “previously diagnosed” group were recruited from a cohort of HIV-infected patients. The “newly diagnosed” and the “suspected” groups were recruited from the outpatients and grouped according to their confirmatory test results. The the exploratory factor analysis was firstly applied to capture the latent structure of participants’ response to the questionnaire about monkeypox. The component and factor scores were compared between groups using the Kruskal-Wallis H tests. The chi-square test was then used to assess the difference of mpox vaccination willingness between MSM and non-MSM in each group. Finally, multivariate logistic regression analysis was performed to identify the determinants of vaccination willingness.

**Results:**

A total of 481 males were included in the final analysis. The results revealed that there was a gap in knowledge about monkeypox between the three participant groups. The vaccination willingness rate of HIV-infected participants was above 90%, while the rate in the HIV-suspected group was 72.60%. Multivariate logistic regression analysis revealed that the previously diagnosed group (adjusted odds ratio [aOR] = 0.314, 95% confidence interval [CI]: 0.105–0.940) and the suspected group (aOR = 0.111, 95% CI: 0.034–0.363) had a lower level of vaccination willingness and they were referred to the newly diagnosed group. Participants in the age groups ranging 25–34 (aOR = 0.287, 95% CI: 0.086–0.959) and 35–44 (aOR = 0.136, 95% CI: 0.039–0.478) years showed a lower level of vaccination willingness, referred to the 15–24 year age group. A better knowledge about monkeypox was associated with a higher level of vaccination willingness (aOR = 1.701, 95% CI: 1.165–2.483). Additionally, a considerable percentage of heterosexual individuals in each group indicated their acceptance of monkeypox vaccines.

**Conclusion:**

An overall high level of vaccination willingness was observed among HIV-infected and-suspected male individuals with disparities noted among those with different HIV infection status, knowledge levels of monkeypox, and age. Addressing the existing knowledge gap and engaging people with persistent risks—regardless of their sexual orientation—for a timely HIV diagnosis may facilitate vaccine-based mitigation measures against monkeypox.

## 1 Introduction

Monkeypox (mpox) is caused by an orthopoxvirus which is similar to smallpox. The virus is symptomatic in the majority of the cases and is characterized by a vesicular rash (1, 2). Prior to 2022, the documented mpox cases outside of Africa had a history of either traveling to the endemic region or having contact with infected animals (2, 3), with no or very limited subsequent human-to-human transmission. However, an unexpected mpox pandemic occurred in 2022 and then spread worldwide rapidly within a year. During the 2022–2023 mpox pandemic, men under the category ‘men who have sex with men’ (MSM) were disproportionately affected, whereas male-to-male sexual contacts were not seen as a dominant transmission route of mpox prior to 2022 (2).

Worldwide, MSM are also at high risk for human immunodeficiency virus (HIV) infection, with acquisition risk 26 times higher than the general population (4). Literature from multiple countries revealed that approximately 40% of mpox cases tested positive for HIV in the recent wave of mpox pandemic (5), and the proportion that HIV-positive males considered in the reported mpox cases was higher than the prevalence of HIV in MSM, implying that people living with HIV (PLHIV) were overrepresented among mpox cases (6–8). Apart from the overlapping at-risk population and a high prevalence of HIV co-infection, mpox also intersected with HIV in clinic treatment (5). For individuals with uncontrolled HIV viral loads, mpox could present a more severe or chronic illness (5, 9). Furthermore, PLHIV coinfected with mpox are more likely subject to dual stigma and higher levels of stress, which may worsen their mental health and clinical outcomes, and impede them from accessing mpox testing, treatment, and vaccination (10–13).

Unfortunately, the current public reporting on mpox has reinforced the stereotypes of “homosexual infection” and exacerbated information barriers (10, 11, 14). Lessons should be learned from HIV/AIDS which is also labeled as a “gay infection” in the early stages. More recently, the epidemiology of HIV infection has changed, and sexual transmission through heterosexuality has begun to predominate in some parts of China. The latest reports on the 2024 outbreak of mpox have shown a similar sign: a broader demographic was affected *via* heterosexual intimate or sexual contact (15). Therefore, it is to be emphasized that the risk of contracting mpox is not limited to MSM, and any person with multiple or new sexual partners is also at risk (16). Without efficient prevention and control measures, there is a possibility of a sustained spillover of the mpox epidemic to the general population, affecting vulnerable groups, such as untreated PLHIV, older adults, immunocompromised individuals, young children, and pregnant women (17, 18).

Considering the aforementioned information, PLHIV and key populations at increased risk of HIV/sexually transmitted infections (STIs) should be valued in preventing mpox virus spillovers, and opportunities should be identified to mitigate the unfavorable impacts of the mpox epidemic on their health (19). First and foremost, promoting an unbiased understanding of mpox infection among the stakeholders can reduce panic and motivate them to adopt preventive behaviors during the epidemic (20). More importantly, pre-exposure prophylactic vaccination should be encouraged for eligible populations to curtail the further spread of mpox (21). Although, currently, there is no mpox-specific vaccine, various generations of smallpox vaccines have been used to protect individuals against mpox due to their cross-immunity to mpox (22). With two doses on a 4-week schedule, the third generation of Modified Vaccinia Ankara live-attenuated vaccine, Bavarian Nordic A/S, Denmark is predicted to provide 71.8% protection against mpox after 2 years (22). MVA-BN shows promising safety and efficacy for PLHIV and is recommended for mpox prevention in many countries, including the UK, Europe, and the USA (18, 23, 56–58). Currently, mpox vaccines are not available in China. Still, it is worth studying vaccination willingness and its determinants among potential target populations to better prepare for the vaccine roll-out in the future. In China, the majority of the existing studies have focused on the vaccination willingness of MSM (with and/or without HIV) on a self-reported basis (24–28); information is limited about people infected with and/or suspected of HIV regardless of gender or sexual orientation. In the context of highly interconnected sexual networks shared by the transmission of HIV/STIs and mpox, louder voices are evident that mpox vaccination should be accessible for anyone who can benefit from it, with a diversity of gender and sexual identity, to achieve a substantial vaccination coverage for cost-effectively decreasing the spread of mpox (18, 19, 29–31). This primary study extended the scope of discussion to HIV-diagnosed and-suspected males without restrictions on self-reported sexual orientation, which brings out two significant tasks: investigating the knowledge gap of mpox and exploring the potential influencing factors of vaccination willingness against mpox among the populations of interest. We foresee this study can update the baseline for addressing the potential disparities in mpox-related health education and the upcoming roll-out of mpox vaccines in China.

## 2 Methods

### 2.1 Design

**Figure 1:**
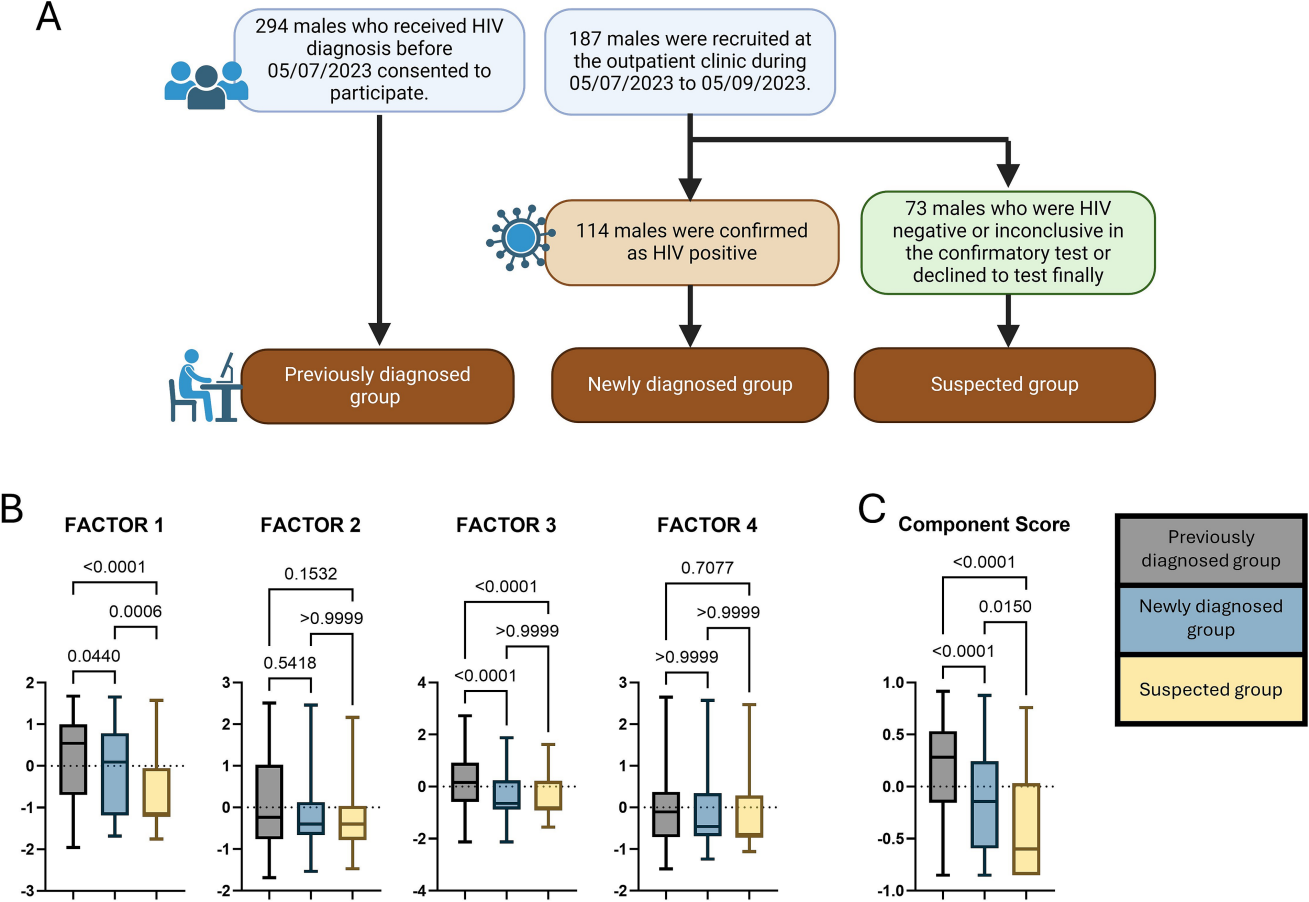
**(A)** The workflow of the participant recruitment; **(B,C)** The comparisons of the component score and the factor scores between participant groups. The *p*-values were acquired by the Kruskal–Wallis H tests.

This study was conducted in Changsha, a provincial city in China with a population of 1,042 million. It started on 5 July 2023 (briefly after the official announcement of the first mpox case in Changsha) and ended on 5 September 2023. As shown in Figure 1A, we adopted the convenience sampling method combining online and field recruitment. Any confirmed and probable cases of mpox were excluded from the recruitment. The first source was obtained through online interviews from a cohort of PLHIV who had received the HIV case management jointly initiated by the local Centers for Disease Control and Prevention (CDC) and antiretroviral therapy (ART) clinics. The CDC interviewers ensured the eligibility criteria by checking digital health records in the recent follow-up visits, including males of age 15 years and above, CD4 cell counts greater than 350/μl, and not inpatients. Finally, 294 people submitted the survey and were labelled a previously diagnosed group. The remainder of the participants were recruited from the outpatient clinic in the municipal centre of the CDC (where a designated HIV confirmatory laboratory is located, offering free confirmatory antibody tests to people with possible HIV exposure). During the data collection period, all male visitors (of age 15 years and above) who consulted about an HIV antibody test were invited to participate. A total of 187 males consented to fill out the survey before taking a test. According to Chinese Guidelines for the Diagnosis and Treatment of HIV/AIDS, the reactive samples in screen tests (test 1 [T1]) are not reported as positive and should be subjected to the following: retesting (test 2 [T2]) and further supplementary testing (test 3 [T3]) before being diagnosed as HIV positive (32). The test takers were further divided into the newly diagnosed (114 people, HIV positive in T3) and the suspected (73 people, HIV negative in T1/T2/T3 or inconclusive in T3 or declined to test finally) groups according to their test results.

### 2.2 Ethical consideration

The study protocol was approved by the Ethics Committee of the Municipal Changsha Center for Disease Control and Prevention (CSCDC). Before the survey, the interviewers explained to the participants about the study’s aims, contents, the potential benefits and dangers of participating in the study, and the participants’ rights. This information was prompted at the beginning of the digitalised questionnaire to ensure that potential participants were sufficiently informed, and the survey submission was regarded as implied consent to participate. The submitted questionnaires, which could be exclusively accessed by the data analyst, were anonymised and saved in the digital storage device secured by a password.

### 2.3 Statistical analysis

The participants used smartphones to access the digitalised survey comprised of three parts: sociodemographics; mpox awareness and related knowledge; sexualities; and mpox vaccination willingness. The instrument for evaluating mpox-related knowledge is a 15-item questionnaire (for details, see Table 1). For each item, the response of “yes” was scored as 1, and the reactions of “no” and “I do not know” were scored as 0. The item scores were added to give a sum ranging from 0 to 15. A training dataset on 100 respondents indicated the feasibility of the factor analysis and an overall Cronbach’s *α* of 0.845 (33). In the formal survey, the exploratory factor analysis (EFA) helped us gather valuable information about the interrelationships of the instrument items (34). Kaiser’s criterion and scree plot were examined to determine the number of factors to be extracted. The varimax orthogonal factor rotation was used to minimize the number of variables with high loadings on each factor. The Kruskal–Wallis H tests were applied to compare the component and factor scores between different participant groups. The chi-square test was used to assess the difference in mpox vaccination willingness between MSM and non-MSM in each group. The variance inflation factor (VIF) was introduced as a multicollinearity diagnosis for the variables to be studied, and we adopted a cutoff value of VIF < 2.5 for selecting variables to enter the logistic regression analysis (35). Finally, multivariate logistic regression was performed to identify determinants of vaccination willingness. All *p*-values were set at <0.05 for statistical significance. SPSS Version 29.0, IBM Corporation, USA. GraphPad Prism Version 9.5.1, GraphPad Software, LLC, USA.

**Table 1:** The matrix of factor loadings, proportion variance and cumulative variance after orthogonal rotation^1^.

Items	Factor 1	Factor 2	Factor 3	Factor 4
1. People are generally susceptible to mpox.	0.053	−0.077	**0.689**	0.036
2. Mild mpox cases can recover without treatment.	0.096	0.046	−0.116	**0.851**
3. One encounter with mpox is followed by lifelong immunity.	0.076	0.105	0.351	**0.642**
4. Mpox can cause mouth sores.	0.150	**0.764**	0.078	0.096
5. Mpox can cause a rash located on the face, chest or palms.	**0.526**	0.408	0.266	0.181
6. Mpox can cause a rash located on the genital or anal areas.	0.195	**0.779**	0.189	−0.047
7. Mpox can cause swollen lymph nodes.	0.198	**0.750**	0.075	0.116
8. People can get mpox by having sex with an infected person.	**0.600**	0.245	0.375	0.191
9. People can get mpox by prolonged face-to-face contact with an infected person.	0.297	0.334	**0.533**	0.235
10. People can get mpox by touching objects that have been used by an infected person.	0.229	0.389	**0.688**	−0.065
11. People can get mpox by close contact with an infected animal.	0.375	0.259	**0.591**	0.071
12. People with weakened immune systems are at higher risk of mpox.	**0.715**	0.145	0.203	0.111
13. People with HIV infection are at higher risk of mpox.	**0.837**	0.160	0.098	0.036
14. Men who have sex with men are at higher risk of mpox.	**0.874**	0.147	0.097	0.023
15. Sex workers are at higher risk of mpox.	**0.833**	0.160	0.134	0.020
SS loadings^2^	3.704	2.421	2.055	1.311
Proportion Var (%)^3^	24.70	16.14	13.70	8.74
Cumulative Var (%)^4^	24.70	40.83	54.54	63.28

^1The 4-factor model reflected an acceptable internal structure according to a factor loading threshold value of 0.4. The bold values are the questionnaire items with a loading above 0.4 on a particular factor, suggesting that the information given by them is substantially influenced by the identified factors.^

^2The normalized variance values associated with factors.^

^3The degree to which each factor explains the whole variance.^

^4The cumulative degree to which factors explain.^

## 3 Results

Sociodemographic information is listed in Table 2. The majority of participants in each group were those aged below 35 years, held a college degree or higher, were employed full-time, and were not married. The answers to five sexuality questions are listed in Table 2. MSM (those whose sexual orientation was reported as gay and bisexual) were responsible for a larger part either in the previously diagnosed (84.70%) or newly diagnosed (73.68%) groups, contrary to the suspected group where heterosexual men predominated (69.86%). Males who had ever used condoms inconsistently in the preceding year made up 78.07% of the newly diagnosed group, much higher than the percentages of the previously diagnosed (44.90%) and the suspected (34.25%) groups. Over one-third of the previously diagnosed and newly diagnosed groups had experienced chemsex (including both legal and illegal use of sexual stimulants, such as Viagra, Cialis, rush poppers, and amyl nitrite) in the past year, and more than 20% of them had same-sex behavior in the past 1 month. Meanwhile, recent casual sex (including commercial sex and group sex) saw the highest level among the suspected ones (21.92%).

**Table 2:** Sociodemographic and mpox-related characteristics.

Variables	Previously diagnosed group *N* (%)	Newly diagnosed group *N* (%)	Suspected group *N* (%)
Age category (years)			
15 ~ 24	68 (23.13)	40 (35.09)	22 (30.14)
25 ~ 34	122 (41.50)	38 (33.33)	22 (30.14)
35 ~ 44	70 (23.81)	19 (16.67)	14 (19.18)
45 or above	34 (11.56)	17 (14.91)	15 (20.55)
Highest education level attained			
Junior high or below	25 (8.50)	16 (14.04)	15 (20.55)
Senior high or equivalent	53 (18.03)	25 (21.93)	16 (21.92)
Professional training college	87 (29.59)	36 (31.58)	17 (23.29)
Bachelor’s degree or above	166 (43.88)	37 (32.46)	25 (34.25)
Marital status			
Married	55 (18.71)	26 (22.81)	29 (39.73)
Unmarried, divorced, or widowed	239 (81.29)	88 (77.19)	44 (60.27)
Current employment status			
Full-time	171 (58.16)	46 (40.35)	35 (47.95)
Part-time	59 (20.07)	28 (24.56)	21 (28.77)
Student, unemployed, or retired	64 (21.77)	40 (35.09)	17 (23.29)
Sexual orientation			
Gay	202 (68.71)	49 (42.98)	12 (16.44)
Bisexual	47 (15.99)	35 (30.70)	10 (13.70)
Heterosexual	45 (15.31)	30 (26.32)	51 (69.86)
Inconsistent condom use in the past 1 year			
Yes	132 (44.90)	89 (78.07)	25 (34.25)
No	162 (55.10)	25 (21.93)	48 (67.75)
Chemsex (such as Viagra, Cialis, rush poppers, and amyl nitrite) in the past 1 year			
Yes	107 (36.39)	39 (34.21)	21 (28.77)
No	187 (63.61)	75 (65.79)	52 (71.23)
Sex with men in the past 1 month			
Yes	85 (28.91)	26 (22.81)	11 (15.07)
No	209 (71.09)	88 (77.19)	62 (84.93)
Casual sex (including commercial sex and group sex) in the past 1 month			
Yes	22 (7.48)	13 (11.40)	16 (21.92)
No	272 (92.52)	101 (88.60)	57 (78.08)
Being aware of mpox epidemic in China			
Yes	247 (84.01)	77 (67.54)	28 (38.36)
No	47 (15.99)	37 (32.46)	45 (61.64)
Mpox knowledge medium (IQR)	9.0 (6.0–11.0)	6.0 (2.0–9.0)	2.0 (0–7.5)
Self-assessed risk of mpox			
Very low	50 (17.01)	22 (19.30)	25 (34.25)
Low	107 (36.39)	44 (38.60)	31 (42.47)
Moderate	89 (30.27)	30 (26.32)	9 (12.33)
High	35 (11.90)	13 (11.40)	6 (8.22)
Very high	13 (4.42)	5 (4.39)	2 (2.74)
Being willing to get vaccinated if vaccines become available in China			
Yes	269 (91.50)	109 (95.61)	53 (72.60)
No	25 (8.50)	5 (4.39)	20 (27.40)
Sum	294	114	73

IQR, interquartile range.

People with awareness of the mpox epidemic in China had the largest share in the previously diagnosed group, at 84.01%, followed by the newly diagnosed group, at 67.54%, and the suspected group, at 38.36%. People who considered their risk of contracting mpox to be moderate and higher accounted for 46.59 and 42.11% of the previously diagnosed and newly diagnosed HIV-infected persons, respectively. Meanwhile, 23.19% of the participants of the suspected group assessed their infection risk as moderate or higher. Over 90% of HIV-infected persons expressed their vaccination willingness, displaying a higher level than the suspected people, 72.60% of whom opted “yes” to this question. MSM had a stronger vaccination willingness than non-MSM in the newly diagnosed group (98.82% vs. 86.67%, *p* = 0.005). In comparison, no statistical difference was in the previously diagnosed group (91.97% vs. 88.89%, *p* = 0.496) and the suspected group (77.27% vs. 70.60%, *p*-value: 0.557).

Table 1 demonstrates that four main factors were recommended by the scree plot for EFA and explained 63.277% of the variance on instrument items. Factor 1 was responsible for 24.70% of the total variance, containing 6 items, namely, a rash on the face, chest, and palms; the mode of sexual transmission; and the at-risk populations (MSM, PLHIV, sex workers, and immunocompromised people) had higher loadings. Three items on other common symptoms had higher loadings on Factor 2, which explained 16.14% of the total variance. Four items investigating the general susceptibility and the rest of the documented transmission modes contributed to 13.170% of the total variance on Factor 3. Finally, 8.74% of the total variance was seen in Factor 4 where 2 items about the prognosis had the higher loadings.

The component score and the factor scores between participant groups are compared as shown in Figures 1B,C. Statistical significance of the Kruskal–Wallis H tests was found in the scores of the component, Factor 1, and Factor 3 (*p* < 0.001). The median of the component score was highest in the previously diagnosed group, followed by the newly diagnosed and the suspected groups. The three groups had the same ranks in comparing the median of Factor 1 score. In terms of the Factor 3 score, the median of the previously diagnosed group was higher than that of the newly diagnosed and the suspected groups. At the same time, there was no statistical significance between the newly diagnosed and the suspected groups’ participants.

A total of 15 variables, including infected group, age group, education level, marital status, employment status, sexual orientation, condom use, chemsex, recent same-sex behavior, casual sex, self-assessed risk, and Factors 1–4 scores, were examined using the VIF, with the results ranging from 1.041 to 2.298, indicating a tolerant multicollinearity consideration. As such, they all entered multivariate logistic regression. As listed in Table 3, three variables had statistical significance (*p* < 0.05) in the final model: participant group, age category, and mpox knowledge. Referring to the newly diagnosed group, the previously diagnosed group (aOR = 0.314, 95% CI: 0.105–0.940) and the suspected group (aOR = 0.111, 95% CI: 0.034–0.363) had lower odds of vaccination willingness. The participants aged between 25 and 34 (aOR = 0.287, 95% CI: 0.086–0.959) and 35–44 (aOR = 0.136, 95% CI: 0.039–0.478) years had lower odds of vaccination willingness in comparison with the participants in 15–24 age category. A higher score in Factor 1 predicted higher odds of vaccination willingness (aOR = 1.701, 95% CI: 1.165–2.483).

**Table 3:** Multivariate logistic stepwise regression of vaccination willingness^1^.

Variables		*B*	SE	Wald × 2	*p*-value^2^	OR^3^	95% CI^4^	
Participant group	Suspected group	−2.202	0.607	13.174	**0.000**	0.111	0.034	0.363
	Previously diagnosed group	−1.157	0.559	4.289	**0.038**	0.314	0.105	0.940
	Newly diagnosed group (reference)							
Age category (years)	45 years or above	−1.110	0.742	2.239	0.135	0.330	0.077	1.411
	35 ~ 44 years	−1.995	0.642	9.666	**0.002**	0.136	0.039	0.478
	25 ~ 34 years	−1.248	0.615	4.110	**0.043**	0.287	0.086	0.959
	15 ~ 24 years (reference)							
Highest education level attained	Junior high or below	0.155	0.557	0.077	0.781	1.167	0.392	3.478
	Senior high or equivalent	−0.400	0.482	0.689	0.406	0.670	0.260	1.724
	Professional training college	−0.098	0.464	0.045	0.833	0.907	0.365	2.251
	Bachelor’s degree or above (reference)							
Marital status	Married	0.437	0.469	0.868	0.351	1.548	0.617	3.882
	Unmarried, divorced, or widowed (reference.)							
Current employment status	Student, unemployed, or retired	−0.667	0.431	2.395	0.122	0.513	0.220	1.195
	Part-time	0.131	0.423	0.095	0.757	1.139	0.498	2.610
	Full-time (reference)							
Sexual orientation	Heterosexual	−0.462	0.548	0.711	**0.399**	0.630	0.215	1.845
	Bisexual	−0.020	0.522	0.001	**0.969**	0.980	0.352	2.726
	Gay (reference)							
Inconsistent condom use in the past 1 year		0.370	0.354	1.092	**0.296**	1.448	0.723	2.898
Chemsex in the past 1 year		−0.249	0.363	0.469	**0.493**	0.780	0.383	1.589
Sex with men in the past 1 month		−0.456	0.470	0.939	**0.332**	0.634	0.252	1.593
Casual sex in the past 1 month		0.662	0.640	1.068	**0.301**	1.938	0.552	6.798
Self-assessed risk of mpox		0.041	0.170	0.058	**0.810**	1.042	0.747	1.453
Factor scores	Factor 1	0.531	0.193	7.562	**0.006**	1.701	1.165	2.483
	Factor 2	0.252	0.212	1.416	**0.234**	1.287	0.849	1.950
	Factor 3	0.344	0.204	2.849	**0.091**	1.411	0.946	2.105
	Factor 4	0.291	0.202	2.090	**0.148**	1.338	0.902	1.987

SE, standard error.

^1Hosmer and Lemeshow test; p > 0.05.^

^2Bold values indicate statistical significance at p < 0.05.^

^3OR = adjusted odds ratio.^

^495% CI = 95% confidence interval.^

## 4 Discussion

This study provided preliminary insights into the knowledge gap of mpox and the possible determinates of vaccine willingness among males living with or at increased risk of HIV during the 2022–2023 mpox pandemic in Changsha. Significant findings are as follows: (1) over 90% of HIV-infected participants were willing to vaccinate for mpox, with a higher level in the newly diagnosed cases; (2) the vaccination willingness level in the HIV-suspected males was above 70%, lower than that in the newly diagnosed patients; (3) a better grasp of key points concerning mpox knowledge (Factor 1) predicted a higher level of vaccination willingness; (4) participants aged between 25 and 44 years were less likely to accept mpox vaccines than the youngest ones; (5) a considerate percentage of heterosexual persons in each group also expressed their acceptance for mpox vaccines; (6) a disparity of mpox knowledge level existed between the three participant groups.

We found a very high level of willingness to vaccinate against mpox in HIV-infected males regardless of sexual orientation. Our findings are on the upper end of homogeneous studies where the levels of mpox vaccine uptake or vaccination willingness were reported in a range of 56.8 to 91.7% among at-risk populations in China (24–27, 36). In a publication issued by *China CDC Weekly*, 78.9% of the under-treated HIV-infected persons with diverse gender and sexual identity were willing to vaccinate in Beijing (36). In our study, the higher level of willingness rates can be partly ascribed to the male-focused design with recruitment of newly confirmed HIV cases who had not been referred to ART treatment and healthcare. These new cases displayed a stronger vaccination willingness than the previously diagnosed patients in our study. A similar finding from a survey of PLHIV in Washington, DC, USA showed that respondents with a recent HIV diagnosis were more likely to be vaccinated for mpox (29).

We also found that newly diagnosed cases were more likely to be vaccinated for mpox than the suspected group’s participants. A possible reason is that these new HIV-infected persons, compared to the suspected ones, had a higher level of previous engagement in high-risk sexuality (e.g., same-sex behavior, inconsistent condom use, and chemsex), and had been more likely to perceive their seroconversion, making them more worried about their vulnerabilities in the mpox epidemic (12, 29). Similarly, Yuwei Li et al. found that MSM with self-reported HIV infection were more prone to vaccine uptake (27). It was noteworthy that, in China, approximately 30% of HIV-infected people were not knowing their status in China (37). For the HIV-suspected people, some were not entirely excluded from HIV infection considering the antibody-negative “window period” or the depletion of antibodies caused by severe immunosuppression (HIV nucleic acid amplification testing can be recommended in these cases) (38, 39). More crucially, frequent HIV tests may indicate a persistent risk for HIV/STIs as well as mpox infection (30, 40). Moreover, STIs and mpox can afflict users of postexposure prophylaxis (PEP) and pre-exposure prophylaxis (PrEP) despite HIV negativity (41). Healthcare providers should engage at-risk populations to adopt risk reduction behaviors and adhere to HIV and STIs (including mpox) testing for a timely medical care. They should also provide quality information about mpox and vaccination to encourage vaccine acceptance in the future vaccination promotion campaigns.

In addition, our results demonstrated that an increased knowledge of mpox predicted a raised level of vaccination willingness in participants, consistent with previous studies (24, 26, 42, 43). However, the statistical significance was only seen with an increase in Factor 1. The first subdomain had the highest correlations with the updated features of epidemiology in the 2022–2023 mpox pandemic. Since mpox has intersected with HIV, according to multiple scientific research reports; public health guidance and messaging; and social media (5, 6, 8, 10, 14, 40), MSM, PLHIV, sex workers, and immunocompromised people are recommended to vaccinate against mpox in multiple regions (13). It is not surprising that an improved knowledge of the intersection between HIV and mpox can make people living with or at risk of HIV better understand the importance of vaccinating for mpox.

Furthermore, we found that willingness to get vaccinated was related to age category, which aligns with several publications (26, 29, 42). However, our analysis did not support a statistical significance in terms of educational levels, sexual orientation/identities, high-risk sexual practices, and perceived risks of mpox infection, which had been reported as the determinants of mpox vaccine uptake or vaccination willingness among at-risk populations in other homogenous studies (25, 29–31, 44). Nevertheless, these studies mainly targeted MSM with a self-reported nature and the results varied across regions and survey periods. We surveyed HIV-infected and-suspected males without restrictions on self-reported sexual orientations and found that a considerable number of heterosexual men in each participant group also expressed their willingness to get inoculated. There was a possibility that the intersection of mpox and HIV raised a health concern among heterosexual persons who had multiple sex partners, making them feel a need to receive mpox vaccines. Meanwhile, we should also consider the high occurrence of MSM’s concealing same-sex behaviors to healthcare providers in China (45, 46). Existing evidence suggested that concealers were more likely to be MSM who were less experienced in HIV testing, had lower self-perceived risk of HIV infection, and had not received HIV-related medical care (46–48). Notably, closeting about sexual orientation can undermine healthcare service utilization, such as STI screening, vaccine uptake, and preventive information seeking (11, 45). Therefore, we believe that the expanded vaccine eligibility for both HIV-infected and-suspected persons inclusive of diverse sexual orientation should be considered and carefully assessed in the development of the vaccination and immunization guidelines for fostering high efficacy of mitigation measures against mpox (29).

The previously diagnosed HIV-infected persons had a better understanding of mpox, including the highest awareness rate of the domestic mpox pandemic and a similar result was reported in an MSM-targeted study (49). It may be primarily contributed to HIV-related healthcare which offered regular health education on coinfection prevention to the diagnosed patients (49). Moreover, a previous HIV diagnosis may have raised their awareness of HIV-related illness (50, 51) and motivated them to use patient peer networks and search engines to gain mpox knowledge (49). A Baidu-index-based study in mainland China indicated that provinces with higher HIV/AIDS incidence had more online search activity related to mpox (52).

Interestingly, in the EFA of mpox knowledge, a skin rash on the face, chest, and palms was most correlated to the items about the updated epidemiological intersection of HIV and mpox. The correlation may reflect a lag in updating public health guidance and messaging on the changes in clinical manifestations. The most prominent symptom of mpox is the typical vesicular rash, based on which a test for mpox virus is recommended (2). In the records before 2022, mpox rash usually started in the face, hands, or feet before spreading to other parts of the body (1), whereas, in the 2022–2023 mpox pandemic, skin lesions predominantly appear on the genital or perianal area (suggesting direct inoculation in sexual contacts), sometimes without preceding prodrome symptoms, and less typical at the beginning, behaving like an STI (2, 18). Failure to understand that mpox can mimic a common STI (e.g., herpes and syphilis) can pose challenges to timely diagnosis because of the diminished suspicion in self-monitoring (53). Moreover, worries over the visibility of symptoms may increase as skin eruptions are mistakenly believed to appear more often in the exposed parts of the body (11, 12). We believe that filling the gap in clinical presentation in public health guidance and messaging has significant implications for guiding at-risk populations in self-monitoring and reducing worries.

There were several limitations to this study. Firstly, our design used a convenient sampling and failed to survey the previously diagnosed HIV-infected persons who had dropped out from the follow-up visits and individuals who refused to participate. Also, the survey was based on self-reports, so there may be recall bias and underpotted involvement in unsafe sexual practices. In addition, the high-risk behaviors were not assessed by frequency, degree, and subcategory, so we were unable to grade the risk of exposure further. Another limitation involves that the newly diagnosed and the suspected group’s participants were recruited from a single center, which may raise a concern about the generalizability of the results. Nevertheless, the municipal centre of the CDC was one of two health institutions authorised to conduct HIV confirmatory antibody tests in Changsha (the only test which was available free of charge for the participants). Additionally, this survey is observational in nature, so there was no causality established. The survey time was carried out when mpox pandemic hit China; these results may change as the pandemic is controlled. Finally, the self-reported data on other STIs were not included in the design. We made this choice mainly due to two reasons. First, HIV/AIDS is distinct from other STIs in terms of etiology, treatments and management, and long-term effects. In this study, we prioritized the intersection of HIV/AIDS and mpox. Second, HIV/AIDS is a chronic infection without a cure and can be confirmed by laboratory tests; in contrast, the diagnosis of an ongoing STI should consider the clinical manifestations. The municipal centre of the CDC is not qualified to make the diagnosis of other STIs, and we were concerned about the incomparability of the self-reported data on STIs with the laboratory-confirmed HIV infection status.

## 5 Conclusion

This study is an attempt to identify the knowledge gap of mpox and determine the potential factors influencing vaccination willingness against mpox among males living with or at increased risk of HIV amid the 2022–23 mpox pandemic in Changsha. We found an overall high degree of vaccination willingness among HIV-infected and-suspected males. Notwithstanding, the willingness levels varied with respect to their HIV infection status, understanding of the intersection of HIV and mpox, and age. Healthcare providers should engage the people who have unsafe sex for a timely HIV and STI (including mpox) diagnosis and treatment, raise their awareness of behavioral modification, and encourage mpox vaccination acceptance of those with persistent exposure, regardless of sexual orientation. Our findings also highlighted the importance of eliminating the potential knowledge gap of mpox for better guiding the at-risk populations in self-monitoring and reducing worries in the mpox epidemic.

## Data Availability

The raw data supporting the conclusions of this article will be made available by the authors, without undue reservation.
